# Antimicrobial-Resistant Bacterial Populations and Antimicrobial Resistance Genes Obtained from Environments Impacted by Livestock and Municipal Waste

**DOI:** 10.1371/journal.pone.0132586

**Published:** 2015-07-21

**Authors:** Getahun E. Agga, Terrance M. Arthur, Lisa M. Durso, Dayna M. Harhay, John W. Schmidt

**Affiliations:** 1 U.S. Department of Agriculture, Agricultural Research Service, Roman L. Hruska U.S. Meat Animal Research Center, Clay Center, Nebraska, United States of America; 2 U.S. Department of Agriculture, Agricultural Research Service, Agroecosystem Management Research Unit, Lincoln, Nebraska, United States of America; Purdue University, UNITED STATES

## Abstract

This study compared the populations of antimicrobial-resistant bacteria and the repertoire of antimicrobial resistance genes in four environments: effluent of three municipal wastewater treatment facilities, three cattle feedlot runoff catchment ponds, three swine waste lagoons, and two “low impact” environments (an urban lake and a relict prairie). Multiple liquid and solid samples were collected from each environment. The prevalences and concentrations of antimicrobial-resistant (AMR) Gram-negative (*Escherichia coli* and *Salmonella enterica)* and Gram-positive (enterococci) bacteria were determined from individual samples (n = 174). The prevalences of 84 antimicrobial resistance genes in metagenomic DNA isolated from samples pooled (n = 44) by collection date, location, and sample type were determined. The prevalences and concentrations of AMR *E*. *coli* and *Salmonella* were similar among the livestock and municipal sample sources. The levels of erythromycin-resistant enterococci were significantly higher in liquid samples from cattle catchment ponds and swine waste lagoons than in liquid samples from municipal wastewater treatment facilities, but solid samples from these environments did not differ significantly. Similarly, trimethoprim/sulfamethoxazole-resistant *E*. *coli* concentrations were significantly higher in swine liquid than in municipal liquid samples, but there was no difference in solid samples. Multivariate analysis of the distribution of antimicrobial resistance genes using principal coordinate analysis showed distinct clustering of samples with livestock (cattle and swine), low impact environment and municipal samples forming three separate clusters. The numbers of class A beta-lactamase, class C beta-lactamase, and fluoroquinolone resistance genes detected were significantly higher (*P* < 0.05) in municipal samples than in cattle runoff or swine lagoon samples. In conclusion, we report that AMR is a very widespread phenomenon and that similar prevalences and concentrations of antimicrobial-resistant bacteria and antimicrobial resistance genes exist in cattle, human, and swine waste streams, but a higher diversity of antimicrobial resistance genes are present in treated human waste discharged from municipal wastewater treatment plants than in livestock environments.

## Introduction

Antimicrobial resistance (AMR) is a natural and ancient phenomenon that precedes the therapeutic use of antimicrobials in humans [1,2]. Hence, infections involving antimicrobial-resistant bacteria (ARB) were reported shortly after the advent of antimicrobial therapy to treat human disease [3,4]. The increasing occurrence of antimicrobial-resistant human infections has been attributed to the selective pressure exerted by the continuous use of antimicrobials in a variety of applications including human and animal disease therapies, food animal production, and horticulture [5]. This has generated concerns over the potential sources and causes of bacterial resistance and animal agriculture has become a focal point in the search for sources of ARB impacting human health [6,7].

Livestock production impacts the occurrence of AMR through the application of antimicrobials for both therapeutic and prophylactic applications. Numerous studies have found AMR in agricultural settings [8–11], leading some to conclude that animal agriculture is the dominant source of AMR. When AMR is reported in animal agricultural settings without comparison to other environments, there is a false pretense that the identified resistance would not be found in non-agricultural environments and is a direct result of antimicrobial use in an agricultural setting [12]. It has been reported recently that application of manure fertilization to agricultural soil led to a bloom in AMR even though the animals that produced the manure had not been treated with antibiotics [13]. The authors concluded that the manure fertilization allowed for enrichment of resident soil bacteria that harbored AMR elements demonstrating that AMR source attribution is complex.

Because AMR is ubiquitous, we hypothesized that specific ARB and antimicrobial resistance genes would be present in multiple environments. Studies have identified ARB in a variety of habitats including: animal feeding operations [9,14–18], municipal waste streams [19,20], and pristine environments with little to no human impact [21–23]. However, these studies did not compare AMR across these habitats. The objective of this study was to compare, using identical methods, ARB and antimicrobial resistance genes from environments associated with municipal sewage treatment plant effluent, cattle feedlot runoff ponds, swine waste lagoons, and environments with minimal direct fecal impact (an urban lake and a relict prairie).

## Materials and Methods

### Ethics Statement

Permission was obtained from private landowners or municipalities for entry and collection at all sample locations. This study did not involve endangered or protected species.

### Sample collection

A total of 174 samples were collected from the effluent of three municipal wastewater treatment facilities (municipal), three cattle feedlot runoff catchment ponds (cattle), three swine waste lagoons (swine), and two environments (an urban lake and a relict prairie) with minimal direct fecal impact by human or livestock fecal waste (low impact). All sampling sites were located in central and eastern portions of Nebraska. Each site was visited twice, once in either July or August 2013 and once in December 2013. All three municipal wastewater treatment facilities utilized some form of disinfection (sodium hypochlorite addition or UV irradiation) for the liquid effluent from April 1 to October 31. The sampling scheme was designed to have one sample period when disinfection was ongoing and one without disinfection. During each visit four liquid and four solid samples were collected, except during the July/August visit to a municipal wastewater treatment facility when only two solid samples were obtained.

Liquid samples (20-ml) consisted of water-sediment slurry. For municipal sites, liquid samples were collected at the location of discharge into the environment. The collection of solid samples (10 g) varied by site, but all solid samples were obtained from material post treatment and was destined for release to the environment. Two municipal sites disposed of dewatered solids in municipal landfills. The third municipal site did not remove water from the solids, which were disposed of by injection into agricultural soil. Each of the examined municipal sites was the sole facility for municipalities with populations between 20,000 and 60,000. Collection of solid samples at the cattle feedlots utilized manure storage piles if available, otherwise samples of pen surface material were collected. Feedlot populations ranged from 10,000 to 50,000 head of cattle. In swine production, solid waste is flushed from the production housing with the liquid waste, both flowing into a lagoon. As such, solids samples were collected around the edge of each lagoon. Swine populations associated with the examined lagoons ranged from 150 to 2500 sows with associated piglet litters. For the December sampling of the prairie, the pond had dried so only sediment samples could be collected instead of a liquid-sediment slurry.

Individual samples (n = 174) were processed by traditional culture techniques for antimicrobial-resistant Gram-negative (*E*. *coli* and *Salmonella*) and Gram-positive (enterococci) bacteria to determine prevalence and to enumerate resistant strains. In addition, samples from each sampling day were pooled (n = 44) by location and sample type for analysis to identify genetic determinants of resistance from the entire bacterial population, the vast majority of which are not amenable to laboratory culture.

### Antimicrobial resistance analyses

The bacterial analyses described herein pertain to common foodborne bacteria resistant to critically important classes of antimicrobials as categorized in Guidance 152 (*Evaluating the Safety of Antimicrobial New Animal Drugs with Regard to Their Microbiological Effects on Bacteria of Human Health Concern*) set forth by the U.S. Food and Drug Administration [24]. The following bacteria were the subjects of investigation in this project: 3rd-generation cephalosporin-resistant (3GC^r^) *E*. *coli*; trimethoprim-sulfamethoxazole-resistant (COT^r^) *E*. *coli*; 3GC^r^ non-typhoidal *Salmonella enterica* (non-typhoidal *Salmonella* will hereafter be referred to as *Salmonella*); nalidixic acid-resistant (NAL^r^) *Salmonella*; and erythromycin-resistant (ERY^r^) *Enterococcus* spp. Each of these antimicrobial resistance classes has been categorized as high priority, critically important by the World Health Organization, with the exception of COT^r^, which has been designated highly important [25]. The genes most commonly identified as encoding resistance to each of the four antimicrobial classes investigated in culture-based portion of this project have been associated with mobile genetic elements, such as plasmids, transposons, and integrons, which are known to be transferred horizontally both inter- and intra-species [26–29]. Thus, the genes encoding resistance to these classes can be harbored in species other than the three common foodborne bacteria (*Enterococcus* spp., *E*. *coli*, and *Salmonella*).

### Bacterial enumeration

After transport to the lab, liquid samples were vigorously vortexed and 50 μl were spiral plated onto different culture media with and without antimicrobials for the enumeration of *E*. *coli*, *Salmonella enterica* and *Enterococcus* species (S1 and S2 Figs). For the purposes of this report the term “generic” will indicate that the bacterial species was isolated from media that did not contain antimicrobials of interest and were not isolated based on any specific resistance. For the enumeration of generic *E*. *coli*, 3GC^r^ *E*. *coli* and COT^r^ *E*. *coli*, CHROMagar *E*. *coli* (DRG International, Mountainside, NJ) was used with no additional antimicrobial (CEC), with 2 mg/liter of cefotaxime (Sigma, St. Louis, MO) (CEC+CTX), or with 4 mg/liter trimethoprim and 76 mg/liter sulfamethoxazole (Sigma) (CEC+COT), respectively [14]. For *Salmonella* enumeration, samples were plated onto xylose lysine deoxycholate (XLD) agar (Remel, Lenexa, MO) plus 4.6 ml/liter tergitol, 15 mg/liter novobiocin and 5 mg/liter cefesulodin (XLD_tnc_) [30]. XLD agar plus 2 mg/liter cefotaxime (XLD+CTX) and XLD agar plus 32 mg/liter nalidixic acid (XLD+NAL) were utilized for growth of 3GC^r^ *Salmonella* spp.; and NAL^r^ *Salmonella* spp., respectively. For the enumeration of enterococci, Slanetz and Bartley medium agar (ThermoFisher, St. Louis, MO) plates (SBM) were used. SBM agar plus 8 mg/liter erythromycin plates (SBM+ERY) were used for erythromycin-resistant (ERY^r^) *Enterococcus* spp. CEC+CTX, CEC+COT, XLD_tnc_, XLD+CTX, and XLD+NAL plates were incubated at 37°C overnight and the SBM and SBM+ERY plates were incubated at 35°C for 4 h then at 44°C for 48 h. For bacterial enumeration from the solid samples, 10 g of the solid matter was added to 90 ml of tryptic soy broth [TSB, Difco, Becton Dickinson] with phosphate buffer [TSB+PO_4_; 30 g of TSB, 2.31 g of KH_2_PO_4_, and 12.54 g of K_2_HPO_4_ per liter of solution; Sigma] and 50 μl appropriate dilutions were spiral plated onto CEC, CEC+CTX, CEC+COT, XLD_tnc_, XLD+CTX, XLD+NAL, SBM, and SBM+ERY (S2 Fig).

### Bacterial prevalence

For the determination of bacterial prevalence, pre-enrichment cultures were prepared by adding 20 ml of the liquid samples to 80 ml of TSB-PO_4_ and 10 g of the solid matter to 90 ml of TSB-PO_4_ [31]. Pre-enrichment broths were incubated at 25°C for 2 h then at 42°C for 6 h, and then held at 4°C until processed the next day. For the enrichment of *Salmonella*, a 1-ml aliquot of the enriched cultures was mixed with 20 μl of *Salmonella* specific immunomagnetic separation beads (Dynal, Lake Success, NY). *Salmonella* was then eluted into 3 ml of Rappaport Vassiliadis soy peptone broth (RVS: Remel) and incubated at 42°C for 18 h [32]. For *E*. *coli*, 0.5 ml of the enriched culture was inoculated to 2.5 ml of MacConkey broth (MCB, Becton, Dickinson and Company, Franklin Lakes, NJ), MCB supplemented with 2.4 mg/liter cefotaxime (MCB+CTX), and MCB supplemented with 4.8 mg/liter trimethoprim and 91.2 mg/liter sulfamethoxazole (MCB+COT) and incubated at 42°C for 18 h [14]. For enterococci, 0.5 ml of the enriched culture was transferred to 2.5 ml of Enterococcosel broth (ECB, Becton, Dickinson and Company) and incubated at 37°C overnight. Following incubation, RVS broth enrichment cultures were swabbed to XLDtnc, XLD+CTX and XLD+NAL plates and incubated at 37°C for 18 h. MCB, MCB+CTX, and MCB+COT *E*. *coli* enrichments were swabbed onto CEC, CEC+CTX and CEC+COT plates, respectively, and incubated at 37°C for 18 h. ECB enterococci enrichments were swabbed onto SBM and SBM+ERY plates and incubated at 35°C for 4 h then at 44°C for 48 h. Up to two bacterial isolates that were presumptively isolated on the basis of characteristic appearance on the respective selective media, were confirmed by using previously described PCR methods for *Salmonella* [33,34], *E*. *coli* [35] and enterococci [36].

### Detection of antimicrobial resistance genes by qPCR array

Forty-four pooled samples were made by combining the individual samples by location, date of sampling, and sample type (solid or liquid). Pooling resulted in 12 samples each for cattle, municipal, and swine and 8 samples for the low impact environment. For pooling, equal volumes of the individual samples to be pooled were combined and centrifuged (10,000 x g for 5 min) to produce a 250 mg pellet for DNA extraction. Total metagenomic DNA was extracted from the pooled samples by using PowerLyzer PowerSoil DNA isolation kit (MO BIO Laboratories, Inc., Carlsbad, CA) according to the manufacturer's instructions. Bead beating by using Bullet Blender Storm 24 (Next Advance, Averill Park, NY) was used for homogenizing the suspension and mechanical disruption of bacterial cells. Microbial DNA qPCR arrays (BAID-1901Z, QIAGEN, Valencia, CA) were used to detect antimicrobial resistance genes according to manufacturer's instructions. The array consisted of 84 resistance genes grouped into aminoglycoside resistance (n = 6), class A (22), class B (9), class C (11) and class D (13) beta-lactamases, fluoroquinolone resistance (10), macrolide-lincosamide-streptogramin_B_ (MLS_B_) resistance (6), multidrug efflux pumps (2), tetracycline efflux pumps (2), vancomycin resistance (2) and one *Staphylococcus aureus* beta-lactam resistance (*mecA*) genes. For each plate the 25 μl reaction volume consisted of 2x microbial qPCR master mix (QIAGEN, Valencia, CA) and 5 ng metagenomic DNA. Plates were incubated in an Applied Biosystems 7500 Fast qPCR thermal cycler (Life Technologies, Grand Island, NY) at 95°C for 10 min followed by 40 cycles of 95°C for 15 sec and 60°C for 2 min. A maximum cycle limit of 34 cycles was used to determine if an AMR gene was present in a sample. Samples that did not meet threshold detection for individual genes by 34 cycles were considered to not harbor those genes. Threshold cycle (C_T_) values were exported to an Excel 2007 data analysis template provided by QIAGEN and were analyzed for the presence or absence of the resistance genes.

### Relative quantification of AMR genes

For relative quantitation, C_T_ values for individual genes were averaged by sample type (cattle, low-impact, municipal, swine). If the average C_T_ value for a specific gene was < 34 for two or more sample types, indicating that the gene was present in the environments, relative quantification was performed. Individual genes were normalized to total bacteria in the sample quantitated with two sets of 16S rRNA primers present in triplicate on each PCR plate.

Calculation of relative abundance used the –ΔΔ*C*T method of Livak and Schmittgen [37].

### Statistical analysis

Arbitrary values were assigned for enumeration and prevalence separately for both sample matrices to overcome the problem of zero bacterial counts based on the lower limit of detections (LLD). For the liquid samples, the theoretical LLD for enumeration and prevalence were 2.00 and -1.30 log_10_ CFU/ml respectively. Accordingly, the arbitrary value for prevalence positive and enumeration negative was 0.35 log_10_ CFU/ml and for prevalence negative and enumeration negative was -2.30 log_10_ CFU/ml. For the solid samples, the theoretical LLD for enumeration and prevalence were 2.30 and -1.00 log_10_ CFU/g respectively. Accordingly, the arbitrary value for prevalence positive and enumeration negative was 0.65 log_10_ CFU/g and the arbitrary value for prevalence negative and enumeration negative was -2.00 log_10_ CFU/g.

Multivariable logistic regression using generalized estimating equations (GEE) model with logit link function was used to investigate the effect of sample sources (environment, cattle, human and swine) on the binary outcomes (e.g. prevalence of bacterial isolates) adjusting for matrix type (liquid and solid) and sampling period (summer and winter) and for clustering effect by location (sampling sites). Similarly, multivariable linear regression using GEE model with identity link function was used to investigate the effect of sample sources on the bacterial counts, expressed as log_10_ colony forming units (CFU/ml for liquid samples and CFU/g for solid samples), adjusting for matrix type and sampling period and also for clustering effect by location. Both the prevalence and bacterial counts data were analyzed by stratifying the data by media type. Bonferroni adjustments were used for all the analyses to account for multiple comparisons and P-values < 0.05 were considered significant. For prevalence values of 0 or 100% the logistic regression models did not converge. In those instances exact binomial 95% confidence intervals were used for pairwise comparisons. These data were analyzed with STATA 13 (StataCorp LP, College Station, Texas). Kruskal-Wallis nonparametric rank test was used to compare the median number of antimicrobial resistance genes detected per sample among the sample sources. Multivariate analysis for antimicrobial resistance gene profiles in each sample were analyzed by principal coordinates analysis (PCoA) with Jaccard similarity index by using Paleontological Statistics (PAST) software package Version 3.0 [38].

## Results

### Prevalence and enumeration

The prevalence and enumeration results for *E*. *coli*, *Salmonella* and *Enterococcus* species are shown in Tables 1 and 2, respectively. Because the concentrations of the vast majority of the low impact environment samples were below the limit of detection by enumeration, those data were not included in Table 2. Similarly, the concentrations of *Salmonella* cultured on media with or without target antimicrobials were below the limit of detection by enumeration for the majority of the samples and as such were not included in Table 2.

**Table 1:** Model adjusted prevalence (%) of *E*. *coli*, *Salmonella* and *Enterococcus* species from cattle (n = 48), low impact environment (n = 32), municipal (n = 46) and swine (n = 48) samples^a^.

	**Environment**			
**Organism**	**Cattle**	**Low-impact**	**Municipal**	**Swine**
*E*. *coli*	93.8^a^	93.8^a^	100^a^	93.8^a^
3GC^r^ *E*. *coli* ^*b*^	79.2^a^	18.8^b^	93.4^a^	72.9^a^
COT^r^ *E*. *coli*	81.3^a^	9.4^b^	100^a^	81.3^a^
*Salmonella*	52.1^a^	3.1^b^	65.7^a^	39.6^a^
3GC^r^ *Salmonella*	35.4^a^	0^b^	14.7^a^ ^,^ ^b^	2.1^b^
NAL^r^ *Salmonella*	0^a^	0^a^	4.3^a^	0^a^
*Enterococcus* species	100^a^	96.9^a^	100^a^	100^a^
ERY^r^ *Enterococcus* species	100^a^	18.8^b^	84.9^a^	91.7^a^

^a^Different superscripts across rows indicate statistically significant (*P* < 0.05) differences between pair of sample sources. Bonferroni adjusted for multiple comparisons. For prevalence values of 0 or 100% the logistic regression models did not converge. In those instances exact binomial 95% confidence intervals were used for pairwise comparisons.

^b^Abbreviations: 3GC^r^ = third generation cephalosporin resistant; COT^r^ = trimethoprim/sulfamethoxazole resistant; NAL^r^ = nalidixic acid resistant; ERY^r^ = erythromycin resistant

**Table 2:** Model adjusted mean log_10_ count of *E*. *coli*, *Salmonella* and *Enterococcus* spp from cattle, municipal, and swine samples^a^.

	**Cattle**	**Municipal**	**Swine**
**Liquid matrix(log CFU/ml)**			
n^b^	24	24	24
*E*. *coli*	2.8^a^	2.7^a^	3.6^a^
3GC^r^ *E*. *coli*	0.7^a^	0.9^a^	1.2^a^
COT^r^ *E*. *coli*	1.3^a^ ^,^ ^b^	0.9^b^	2.0^a^
*Enterococcus* species	3.1^a^	2.1^a^	3.1^a^
ERY^r^ *Enterococcus* species	2.7^a^	0.4^b^	2.4^a^
**Solid matrix (log CFU/g)**			
n	24	22	24
*E*. *coli*	2.4^a^	3.4^a^	1.5^a^
3GC^r^ *E*. *coli*	0.13^a^	1.4^a^	-0.5^a^
COT^r^ *E*. *coli*	0.3^a^	1.9^a^	-0.06^a^
*Enterococcus* species	2.9^a^ ^,^ ^b^	3.3^a^	1.9^b^
ERY^r^ *Enterococcus* species	2.1^a^	2.0^a^	0.8^a^

^a^Different superscripts across rows indicate statistically significant (*P* < 0.05) differences between pair of sample sources. Bonferroni adjusted for multiple comparisons.

^b^Abbreviations: n = number of samples; 3GC^r^ = third generation cephalosporin resistant; COT^r^ = trimethoprim/sulfamethoxazole resistant; ERY^r^ = erythromycin resistant.

The prevalences and concentrations of generic *E*. *coli* were not significantly different (*P* > 0.05) among all of the environments with the exception of the *E*. *coli* concentrations in the low impact environment, which were significantly (*P* < 0.05) lower (≈ 3 logs lower) than livestock or municipal environments. While the prevalences of 3GC^r^ and COT^r^ *E*. *coli* were highest in the municipal environment as compared to the cattle or swine environments, the differences were not statistically significant (*P* > 0.05). Similarly, there were no statistically significant differences (*P* > 0.05) in the concentrations of 3GC^r^ *E*. *coli* obtained from cattle, municipal and swine samples. The COT^r^ concentration in swine liquid samples was significantly higher (*P* < 0.05) than COT^r^ concentrations in municipal liquid samples, but there was no difference in solid samples. 3GC^r^ and COT^r^ *E*. *coli* were commonly found in cattle, human and swine waste samples (all prevalences > 70%), but were obtained at lower frequencies (18.8% and 9.4%, respectively) in the low impact environment samples. In addition, all low impact environment samples had concentrations of 3GC^r^ and COT^r^ *E*. *coli* below the limit of detection for enumeration.

The prevalences of generic *Salmonella* were not significantly different among cattle, municipal, and swine waste samples. *Salmonella* was isolated from only one low impact environment sample. The 3GC^r^ *Salmonella* prevalence among cattle samples was significantly higher (*P* < 0.05) than 3GC^r^ *Salmonella* prevalence in either low impact or swine samples, but not different (*P* > 0.05) from municipal samples. NAL^r^ *Salmonella* was recovered only from the municipal environment as two samples from one municipal environment in the summer were found to be positive.

Enterococci prevalence did not differ (*P* > 0.05) between any environments. The prevalence of ERY^r^ enterococci did not differ (*P* > 0.05) among cattle, human, and swine-associated environments, but was significantly lower (*P* < 0.05) for low impact environments. The concentration of ERY^r^ enterococci in municipal samples as compared to human- or swine-associated samples was significantly (*P* < 0.05) lower for liquid samples, however there was no statistically significant difference (*P* > 0.05) in the solid samples. Similar to generic *E*. *coli* concentrations, the concentration of generic enterococci in the low impact environment was significantly (*P* > 0.05) lower (≈ 2 logs lower) than enterococci concentrations in the other environments.

Sampling period (summer or winter) was not significantly (*P* > 0.05) associated with the prevalence or levels of the bacterial species studied.

### Detection of antimicrobial resistance genes from metagenomic DNA

**Fig 1:**
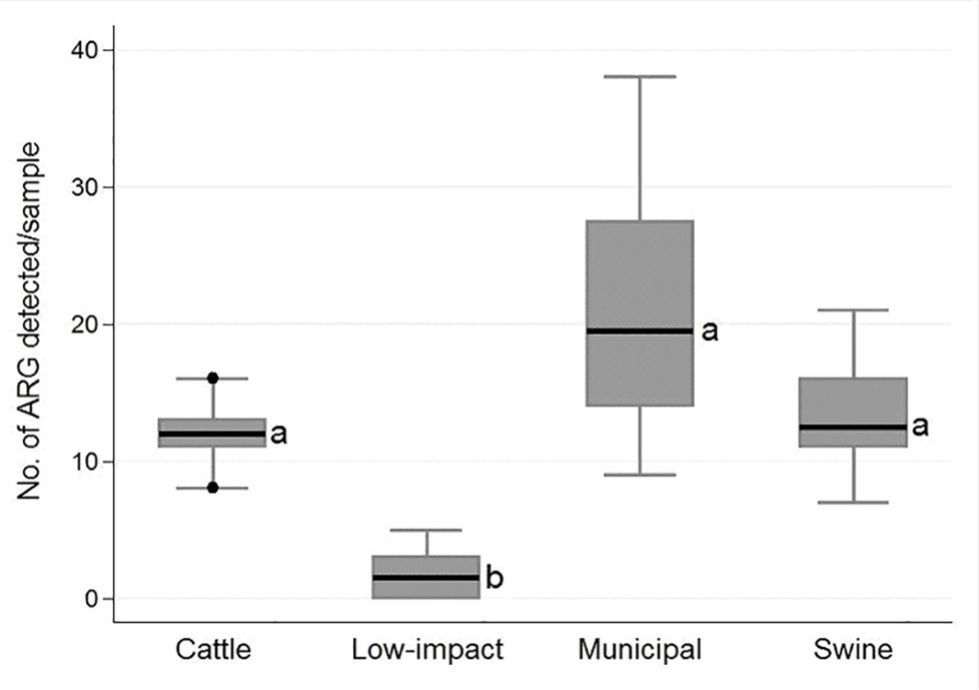
Box plot showing median distribution of antimicrobial resistance genes detected per pooled sample among cattle (n = 12), low impact environment (n = 8), municipal (n = 12) and swine (n = 12) samples. The bold horizontal lines represent the median. The whiskers represent the upper and lower adjacent values. Superscripts have been assigned to the median. Different superscripts indicate statistically significant (P = 0.0001) differences between pairs of sample sources.

A total of 61 out of 84 unique antimicrobial resistance genes were detected from 41 of the 44-pooled samples tested (Table 3). Three of the eight low impact environment-pooled samples were negative for any of the resistance genes targeted. The top ten most prevalent resistance genes for all pooled samples (occurring in >50% of all samples) were aminoglycosides (*aadA1*) and aminoglycosides/fluoroquinolone resistance (*aac(6')-Ib-cr*), class D beta-lactamases (OXA-2 group and OXA-10 group), MLS_B_ resistance (*ermA*, *ermB*, *ermC*, and *mefA*) and tetracycline efflux genes (*tet*A and *tet*B). The median number of antimicrobial resistance genes detected per pooled sample was 19.5 (range: 9‒38), 12.5 (range: 7‒21), 12 (range: 8‒16), and 1.5 (range: 0‒5) in the municipal, swine, cattle, and low impact environment samples, respectively. The median number of antimicrobial resistance genes detected per sample from low impact environments was significantly (*P* = 0.0001) lower than the corresponding median values among livestock and municipal samples (Fig 1)

**Table 3:** Number of cattle (n = 12), low impact environment (n = 8), municipal (n = 12) and swine (n = 12) pooled samples harboring specific antimicrobial resistance genes.

**Antibiotic resistance classes**	**Genes**	**Cattle**	**Low-impact**	**Municipal**	**Swine**
**Aminoglycoside resistance**	*aac*C1	3	1	12	1
	*aac*C2	7		6	5
	*aac*C4	5		1	11
	*aad*A1	12		12	12
	*aph*A6			3	6
**Class A beta-lactamases**	BES-1			4	
	CTX-M-1 Group			5	
	CTX-M-9 Group			2	
	GES			12	2
	IMI & NMC-A		1		
	KPC			3	
	Per-1 group			2	
	Per-2 group		1	1	2
	SFO-1			1	1
	SHV			6	
	SHV(156D)				1
	SHV(156G)	2		8	2
	SHV(238G240E)	1		7	1
	SHV(238S240K)			1	1
	TLA-1	1		8	
	VEB	5		10	4
**Class B beta-lactamases**	IMP-2 group		1		
	IMP-5 group	2			1
	VIM-1 group			2	
**Class C beta-lactamases**	ACC-3				1
	ACT 5/7 group	1		2	1
	ACT-1 group		1	4	1
	CMY-10 Group			7	
	DHA			1	
	FOX			2	
	LAT			4	
	MIR		1	3	
	MOX			9	
**Class D beta-lactamases**	OXA-10 Group	6		12	10
	OXA-18			1	
	OXA-2 Group	11		12	11
	OXA-23 Group		1		
	OXA-24 Group			4	
	OXA-48 Group			1	
	OXA-50 Group			1	
	OXA-51 Group			1	
	OXA-55	1			
	OXA-58 Group			4	5
**Fluoroquinolone resistance**	AAC(6)-Ib-cr	6		12	11
	QnrA	1			
	QnrB-1 group			3	
	QnrB-4 group			1	
	QnrB-5 group			3	
	QnrB-8 group			1	
	QnrC				1
	QnrD	1		1	
	QnrS			10	
**Macrolide resistance**	*ere*B	9		2	3
	*erm*A	12		3	11
	*erm*B	12	1	12	12
	*erm*C	12		3	12
	*mef*A	12	3	12	12
	*msr*A			2	
**Multidrug resistance efflux pump**	*opr*m			1	
**Tetracycline resistance**	*tet*A	12	1	12	11
	*tet*B	11		3	9

**Fig 2:**
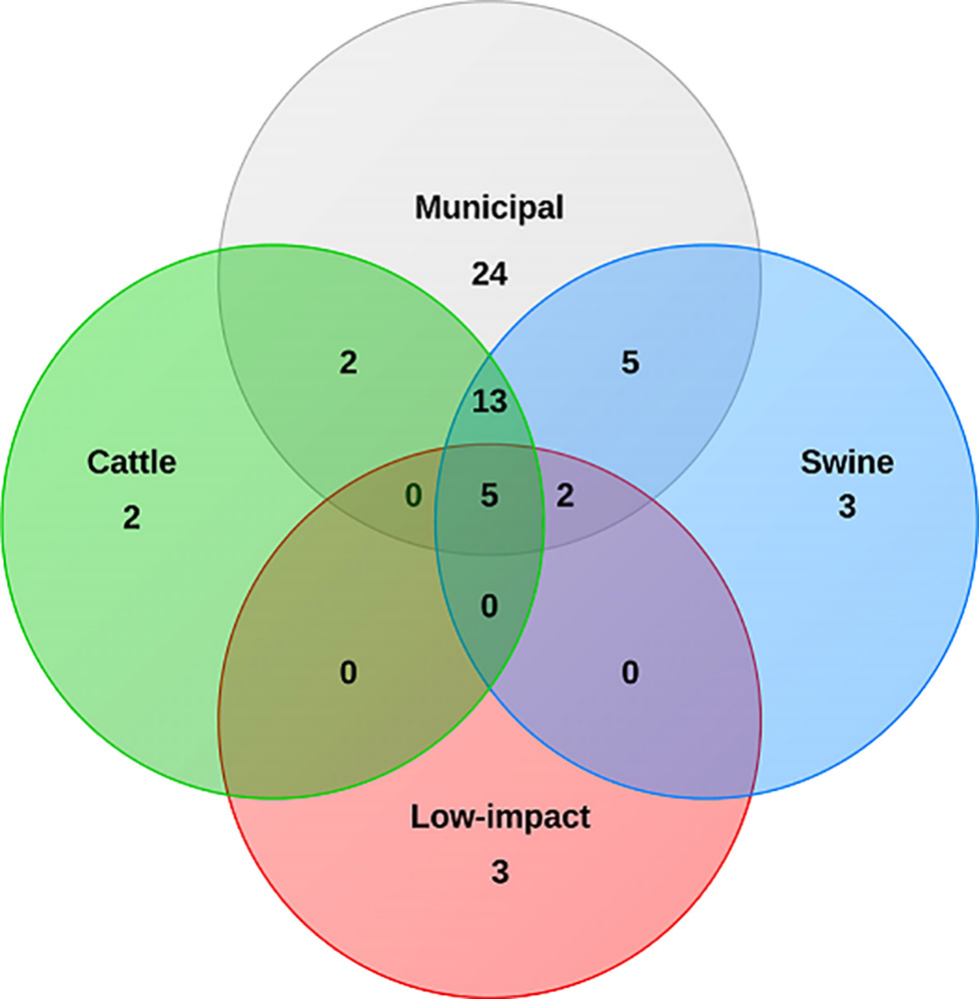
Venn diagram showing the number of specific genes identified by sample source. One gene common to cattle and swine samples and one gene common to low impact and municipal samples were not shown on the Venn diagram. The two genes are common to circles that cannot intersect in this diagram.

When broken down by antibiotic resistance class, the number of class A beta-lactamase, class C beta-lactamase, and fluoroquinolone resistance genes detected from municipal samples were significantly higher (*P* < 0.05) than the number of genes detected from cattle, low impact environment or swine samples. The individual antimicrobial resistance genes detected in samples from municipal samples tended to be more diverse than in samples obtained from other sources with 52, 29, 23 and 11 resistance genes detected from municipal, swine, cattle and low impact environment sources respectively (Table 3, Fig 2).

Four genes (*aacC1*, *ermB*, *mefA*, *tetA*) were shared among all four environments indicating that aminoglycoside, MLS_B_, and tetracycline resistance determinants were present in all environments tested. Other resistance genes were found in multiple environments including 13 shared among cattle, municipal, and swine samples, two shared among municipal, swine, and low impact environment samples, five were shared only between municipal and swine, two shared only between cattle and municipal samples, a single gene each shared only between cattle and swine and only between low impact environment and municipal (Fig 2, Table 3). Twenty-four resistance genes were unique to municipal samples, but only three genes were unique to swine lagoons, three unique to low impact environment samples and two unique to feedlot cattle runoff samples.

The four most frequently observed antimicrobial resistance genes that were unique to the twelve pooled municipal samples were QnrS (detected in 10 of 12, 83%, fluoroquinolone), MOX (75%, class C beta-lactamase), CMY-10 group (58%, class C beta-lactamase) and SHV (50%, class A beta-lactamase) (Table 3). Aminoglycoside resistance gene *aacC1* and class A beta-lactamase gene GES were found in 100% of the pooled municipal samples, but only rarely in other environments. Surprisingly, resistance genes that were unique to the cattle runoff, low impact environment, or swine lagoon samples were observed in only one of the pooled samples from each source. Genes coding for carbapenem-hydrolyzing enzymes (GES, IMI & NMC-A, KPC, IMP-2 group, IMP-5 group, VIM-1 group, OXA-23 Group, OXA-24 Group, OXA-48 Group, OXA-51 Group, and OXA-58 Group) were found predominantly (27 of 40 occurrences) in the municipal environments, but were detected in livestock and low impact samples as well.

**Fig 3:**
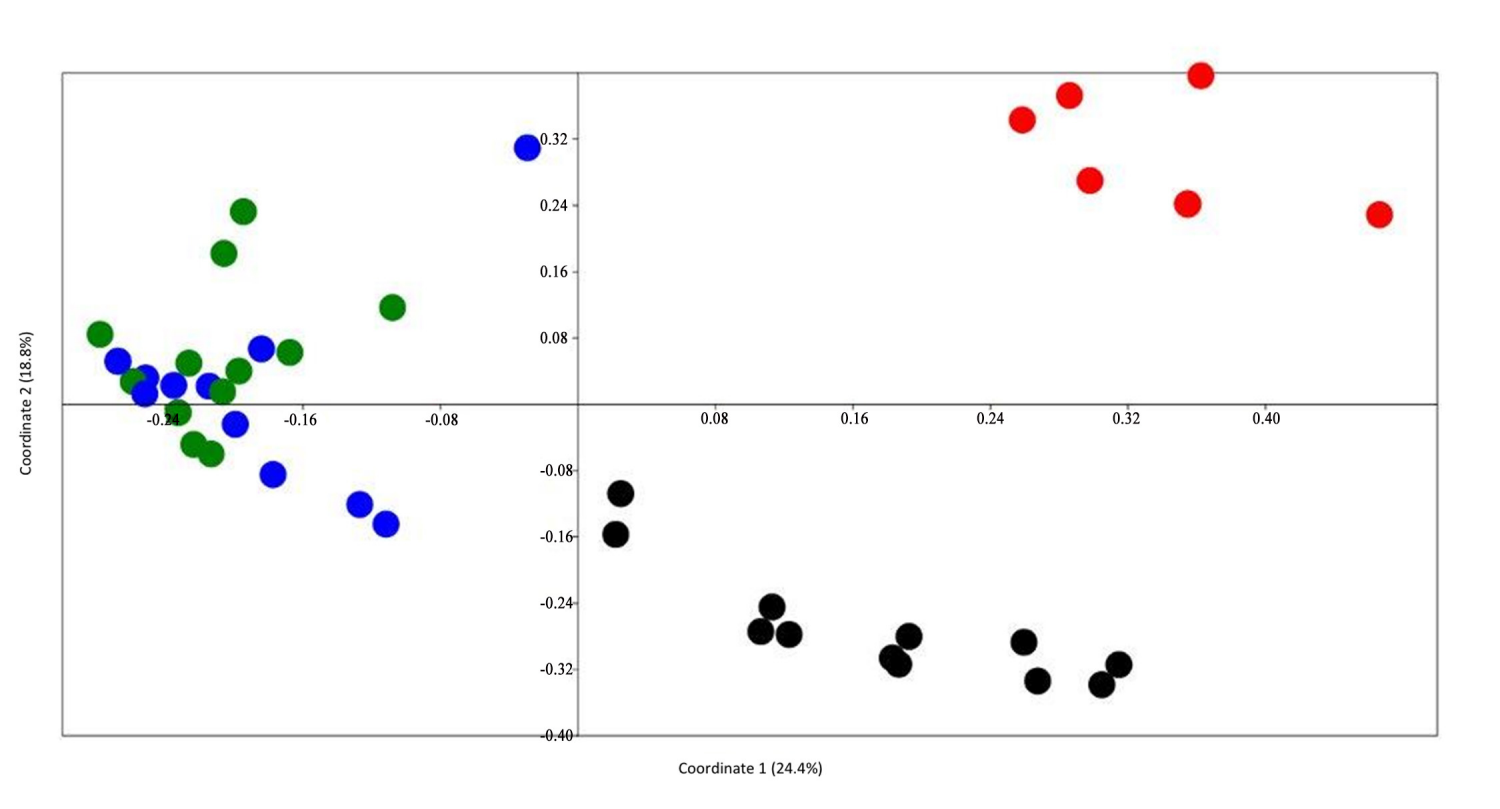
Principal coordinate analysis showing the clustering of antimicrobial resistance genes by livestock, municipal and low impact environmental samples. Antimicrobial resistance genes were detected among cattle (n = 12), low impact environment (n = 8), municipal (n = 12) and swine (n = 12) pooled samples. Data points are colored as follows: green = cattle, red = low impact environment, black = municipal and blue = swine.

A multivariate analysis of the distribution of antimicrobial resistance genes using principal coordinate analysis showed distinct clustering of samples within livestock (cattle and swine), low impact environment and municipal samples forming three separate clusters (Fig 3). Samples within each source (cluster) were more similar to each other with respect to their antimicrobial resistance gene profiles whereas samples from different sources (clusters) were more dissimilar. Municipal and low impact environment samples were not related to each other thus forming two separate clusters. However, cattle and swine samples were closely related to each other forming a third cluster and were not related to either municipal or low impact environment samples.

### Relative quantitation of antimicrobial resistance genes from metagenomic DNA

Only seven AMR genes [AAC(6)-Ib-cr, *aad*A1, OXA-10 group, OXA-2 group, *erm*B, *mef*A, and *tet*A] were common to cattle, municipal and swine samples and had average C_T_ values < 34 (S1 Table). All seven genes were more abundant in municipal samples than either cattle or swine samples. When cattle samples were compared to municipal, four genes [AAC(6)-Ib-cr, OXA-10 group, OXA-2 group, and *erm*B) representing three classes of antimicrobials were at least 10-fold more abundant in the municipal samples than in the cattle samples. The beta lactamase OXA-10 group had the largest disparity being approximately 3 log_10_-fold more abundant in municipal samples than in cattle samples. A similar trend was observed when swine samples were compared to municipal samples (S1 Table).

## Discussion

This study set out to determine the relative contribution of animal agriculture to AMR as compared to the treated effluent from municipal wastewater treatment plants and environments not expected to be impacted by the selective pressure of antimicrobials. Animal agriculture has become a focal point for the spread of AMR primarily based on the amount of antimicrobials used in food animal production [6,7]. It should be noted that one-third of the antimicrobials utilized in food animal production (ionophores) [39] do not have any equivalent drugs used for human therapeutic purposes. Tetracyclines, which make up another 40% of total antimicrobials used in animal agriculture, are not considered a first-line antimicrobial treatment in human medicine [40]. However, there are several antimicrobials administered to food animals that are analogs to human therapeutic compounds and many studies have documented resistance to antimicrobials that are critically important in fighting human disease in samples from food animal production environments [10,11,41–43]. The deficiency in turning complete focus towards animal agriculture based on these previous, isolated studies is that in the absence of rigorous epidemiological tracking data, AMR prevalence results need to be placed into the context of AMR as a whole [44].

AMR is an ancient phenomenon and is present in most environments [1,2,22]. AMR has been a common occurrence long before the clinical use of antimicrobials. It would appear that the presence of AMR is less a function of the ecosystem under study, but rather the methods used to detect AMR. In the current study, 3GC^r^ *E*. *coli*, COT^r^ *E*. *coli*, and ERY^r^ enterococci were isolated from low impact samples collected in environments not expected to have much exposure to ARB populations or antimicrobial selective pressure. These low impact environment samples also were found to contain antimicrobial resistance genes for 7 of the 10 resistance classes that were screened for. In addition, three carbapenemase genes (IMI & NMC-A, IMP-2 group, and OXA-23 group), coding for resistance elements associated with carbapenem-resistant *Enterobacteriaceae*, opportunistic pathogens that are extremely difficult to treat clinically and assigned a threat level of urgent by the Centers for Disease Control (40), were detected in samples obtained from the relict prairie. It is possible that the origins of resistance in these low impact environments could be attributed to the spread of ARB via direct fecal contamination from wildlife or companion animals [45–47] as well as indirect contamination via runoff from weather-related events [48,49]. It may also be that the AMR elements are intrinsic to these environments.

Udikovik-Kolic et al. [13] recently demonstrated an increase in ARB populations in soil fertilized with manure. Surprisingly, the source of ARB was not attributed to the manure, as the cattle that produced the manure had not been treated with antimicrobials. The authors concluded that the increase was due to growth of a resident ARB population in the soil with the manure providing the nutrients and other factors necessary for growth [13]. It should be noted that in the current study ARB were found somewhat frequently (9.4% prevalence of COT^r^ *E*. *coli* and 18.8% prevalence of 3GC^r^ *E*. *coli* and ERY^r^ enterococci) in the low impact environments in spite of the fact that the total bacterial populations, as measured via generic *E*. *coli* and enterococci counts, were 2 to 3 logs lower in the low impact environments compared to the livestock or municipal environments. This implies that if the total bacterial populations in the low impact environments were increased to levels comparable to the livestock and municipal environments, one may observe comparable prevalences of ARB.

Previous studies have reported detection of AMR in environments not considered to be exposed to antimicrobials [50–52]. Miteva et al. [50] recovered several multidrug-resistant psychrophiles from an ice core sample removed from a glacier in Greenland. These microorganisms were believed to be solidified in the ice 120,000 years ago. The authors concluded from this finding that AMR is ubiquitous and not dependent on human application of antibiotics [50]. Brown and Balkwill [51] recovered ARB from deep subsurface sediments, which had not been influenced by surface phenomenon for 3 million years. Most of the isolates recovered in the study were resistant to more than one antimicrobial, with one isolate resistant to eight antimicrobials. Similarly, Bhullar et al. [52] investigated the AMR microbiome of a cave isolated from surface perturbations for over 4 million years. Again, many isolates were multidrug resistant with some isolates resistant to 14 antimicrobials.

The existence of vast ARB populations in the absence of human-applied antimicrobial selective pressure is not confined to soils and sediments. Several studies have reported the presence of ARB in the intestinal tracts of individuals living in remote communities with minimal to no antimicrobial use. Davis and Anandan [53] studied a community in North Borneo and concluded that multiple-antimicrobials resistance elements existed in human populations prior to the introduction of man-made antimicrobials. High prevalences of tetracycline, ampicillin, trimethoprim-sulfamethoxazole, and chloramphenicol-resistant *E*. *coli* were recovered from fecal samples from subjects living in remote communities of Bolivia and Peru [54,55]. Interestingly, the prevalences of COT^r^ *E*. *coli* reported for individuals in these remote communities (50% Bolivia and 67% Peru; [54,55]) with minimal to no antimicrobials exposure were comparable to those seen for the livestock environments (81.3% for both cattle and swine environments) in the current study.

When comparing the low impact environments to the animal agriculture environments for the study described herein, similar types of bacteria and resistance gene classes were observed, however the livestock samples contained higher concentrations of the specific bacterial types and more diversity of ARGs within each resistance class. This was observed to a greater extent when the municipal samples were used in the comparisons. While the solid and liquid effluent samples from the municipal wastewater treatment facilities had comparable prevalences and concentrations of ARB as compared to the livestock samples, municipal samples were the only samples found to harbor NAL^r^ *Salmonella*. In addition, municipal samples contained higher prevalences and more diversity of antimicrobial resistance genes than any of the other sample types. In addition, antimicrobial genes found in multiple environments tended to be in higher abundance in the municipal environments than in livestock environments. This was not unexpected; as many studies have consistently demonstrated that materials discharged from wastewater (WWT) facilities carry high levels of antimicrobial residues and ARB [56–61].

Antimicrobials can enter municipal WWT facilities from various routes [62–64]. One potential route involves antimicrobials and their pharmacologically active metabolites that are excreted from patients following a clinical treatment regimen. Another route comes from improper disposal of unused or expired antimicrobials via discharge to a local sewer system by individuals or institutions. These inputs of antimicrobials to WWT facilities are accompanied by high concentrations of antimicrobial-resistant and susceptible bacteria. Therefore, there can be selective pressure and sufficient antimicrobial resistance genes levels to facilitate amplification of the ARB population in the WWT facility liquid effluent and discarded biosolids [65,66]. Surprisingly, those facilities receiving waste streams from hospitals appear to be no more likely to discharge ARB than WWT facilities that did not receive hospital effluent [67]. One hypothesis put forward to explain this was that the levels of ARB in WWT facilities were already very high, hence additional inputs from hospital waste would not be discernible [67].

It is clear that multiple resistance types are commonly found in many environments. This is perhaps most evident using metagenomic studies. Metagenomic analyses allow for screening of many more resistance elements than traditional bacterial culture by not having to run a separate assay for each resistance to be investigated. Nesme et al. [68] analyzed 71 environments in a metagenomic study for antimicrobial resistance. The environments included Antarctic lakes, the Atlantic ocean, soils from geographically distant regions, and intestinal tract samples from chickens, cows, humans, and mice. The authors found antimicrobial resistance genes determinants in all 71 environments with soil metagenomes harboring the most diverse groups of antimicrobial resistance genes determinants [68]. Another finding from the study was that the antimicrobial resistance genes determinants were clustered by environment. Hierarchical clustering grouped human feces, ocean and soil metagenomes into three distinct clusters by environment [68]. Soil and human feces shared more resistance classes with each other than either did with ocean metagenomes. In the current study, clustering by environment was observed as well. Cattle and swine environments were quite similar based upon antimicrobial resistance gene content, while the municipal and low impact environments were unique. This indicates that ARB populations associated with animal agriculture are distinct from those associated with human activity. A recent study of AR *Salmonella* DT104 isolated from samples of sympatric human and animal populations in Scotland identified that the DT104 strains obtained from each of these two communities were epidemiologically distinguishable [69]. This finding led the authors to conclude that cattle were unlikely to be the major source of resistance diversity for humans and that restricting antimicrobial use in domestic animals in order to curb resistance in humans may not be effective [69].

The use of antimicrobials in food animal production does provide selective pressure for the amplification of AMR, but the impact on human health is difficult to measure. One difficulty in determining the impact of animal agriculture on AMR with regard to human health is that AMR can be thought of as ubiquitous and current tracking methods lack adequate resolution for source attribution. Hence, when ARB are found in a particular environment, conclusions may be formed based on data that were not placed in proper context.

A second issue that complicates the linkage of antimicrobials use in agriculture with human health concerns is that a direct correlation between veterinary antimicrobials usage and AMR in human clinical isolates has not been established. It has been reported from the United Kingdom that trends in AMR characteristics for *Salmonella* isolated from human clinical disease cases in England and Wales did not correspond to fluctuations of veterinary antimicrobials sales in the same regions [70]. The authors noted several divergent trends most notably the increase in fluoroquinolone resistance of *S*. Enteritidis 11% to 26% from 2000 to 2004, while the veterinary sales of fluoroquinolones had dropped by 17% over the same time period. One factor possibly affecting this outcome is that increases in AMR following animal treatment appear to be transient. Several studies have shown that when cattle are given antimicrobials treatments there is an increase in the ARB population, which then wanes shortly after the therapy is completed, returning to baseline population levels [9,71–73]. Cox et al. [71] modeled the *Salmonella Typhimurium* population associated with cattle in England and Wales and observed peaks of resistance in mid-late spring and a lesser peak in late autumn. The authors determined these peaks to be associated with times of calving and animal transport, which would be associated with the main periods of antimicrobials treatments as well. The authors also documented the rapid decrease in resistance during periods where antimicrobials use in cattle was less [71]. Schmidt et al. [9] performed a longitudinal study demonstrating that cattle treatments with ceftiofur led to a transient increase of 3GC^r^ *E*. *coli* shedding following ceftiofur treatment, but quickly thereafter ceftiofur-treated cattle were no more likely than untreated members of the same herd to shed 3GC^r^ *E*. *coli*. Similar findings of transient increases in 3GC^r^ *E*. *coli* populations following ceftiofur treatment of cattle were reported by Lowrance et al. [72] and Singer et al. [73].

In this study we demonstrated that similar levels of ARB and antimicrobial resistance genes can be obtained from livestock and treated human waste when these environments are sampled and analyzed with identical methods and that the diversity of antimicrobial resistance genes is highest in environments associated with treated human waste. In addition, ARB populations and several antimicrobial resistance genes were detected in the low impact environment samples in spite of the fact that the total bacterial populations, as represented by *E*. *coli* and enterococci, in those environments were low. Hence, when assessing risk for development and spread of ARB, a survey of a discrete environment does not provide the necessary context for valid risk assessment.

## Supporting Information

S1 Fig Flow diagram of liquid sample processing. (TIF)

S2 Fig Flow diagram of solid sample processing. (TIF)

S1 Table Relative abundance of antimicrobial resistance (AMR) genes in livestock and municipal environments. The file contains data from relative quantitation of AMR genes. Comparisons were between municipal and cattle samples and municipal and swine samples.
(DOCX)

## References

[1] D'Costa VM, King CE, Kalan L, Morar M, Sung WW, et al (2011) Antibiotic resistance is ancient. Nature 477: 457–461. 10.1038/nature10388 21881561

[2] Wright GD, Poinar H (2012) Antibiotic resistance is ancient: implications for drug discovery. Trends Microbiol 20: 157–159. 10.1016/j.tim.2012.01.002 22284896

[3] Lowbury EJ (1955) Cross-infection of wounds with antibiotic-resistant organisms. Br Med J 1: 985–990. 14363768 10.1136/bmj.1.4920.985 PMC2061717

[4] Kirby WM (1944) Extraction of a Highly Potent Penicillin Inactivator from Penicillin Resistant Staphylococci. Science 99: 452–453. 17798398 10.1126/science.99.2579.452

[5] Davies J, Davies D (2010) Origins and evolution of antibiotic resistance. Microbiol Mol Biol Rev 74: 417–433. 10.1128/MMBR.00016-10 20805405 PMC2937522

[6] Sarmah AK, Meyer MT, Boxall AB (2006) A global perspective on the use, sales, exposure pathways, occurrence, fate and effects of veterinary antibiotics (VAs) in the environment. Chemosphere 65: 725–759. 16677683 10.1016/j.chemosphere.2006.03.026

[7] Aarestrup FM (2005) Veterinary drug usage and antimicrobial resistance in bacteria of animal origin. Basic Clin Pharmacol Toxicol 96: 271–281. 15755309 10.1111/j.1742-7843.2005.pto960401.x

[8] Agga GE, Scott HM, Amachawadi RG, Nagaraja TG, Vinasco J, et al (2014) Effects of chlortetracycline and copper supplementation on antimicrobial resistance of fecal *Escherichia coli* from weaned pigs. Prev Vet Med 114: 231–246. 10.1016/j.prevetmed.2014.02.010 24655578

[9] Schmidt JW, Griffin D, Kuehn LA, Brichta-Harhay DM (2013) Influence of therapeutic ceftiofur treatments of feedlot cattle on fecal and hide prevalences of commensal *Escherichia coli* resistant to expanded-spectrum cephalosporins, and molecular characterization of resistant isolates. Appl Environ Microbiol 79: 2273–2283. 10.1128/AEM.03592-12 23354706 PMC3623230

[10] Kanwar N, Scott HM, Norby B, Loneragan GH, Vinasco J, et al (2013) Effects of ceftiofur and chlortetracycline treatment strategies on antimicrobial susceptibility and on tet(A), tet(B), and bla CMY-2 resistance genes among E. coli isolated from the feces of feedlot cattle. PLoS One 8: e80575 10.1371/journal.pone.0080575 24260423 PMC3834275

[11] Brooks JP, McLaughlin MR (2009) Antibiotic resistant bacterial profiles of anaerobic swine lagoon effluent. J Environ Qual 38: 2431–2437. 10.2134/jeq2008.0471 19875799

[12] Durso LM, Cook KL (2014) Impacts of antibiotic use in agriculture: what are the benefits and risks? Curr Opin Microbiol 19: 37–44. 10.1016/j.mib.2014.05.019 24997398

[13] Udikovic-Kolic N, Wichmann F, Broderick NA, Handelsman J (2014) Bloom of resident antibiotic-resistant bacteria in soil following manure fertilization. Proc Natl Acad Sci U S A. 10.1073/pnas.1409836111 PMC4210343 25288759

[14] Schmidt JW, Agga GE, Bosilevac JM, Brichta-Harhay DM, Shackelford SD, et al (2014) Occurrence of Antimicrobial-Resistant *Escherichia coli* and *Salmonella enterica* in the Beef Cattle Production and Processing Continuum. Appl Environ Microbiol. 10.1128/AEM.03079-14 PMC4277590 25398858

[15] Durso LM, Harhay GP, Bono JL, Smith TP (2011) Virulence-associated and antibiotic resistance genes of microbial populations in cattle feces analyzed using a metagenomic approach. J Microbiol Methods 84: 278–282. 10.1016/j.mimet.2010.12.008 21167876

[16] Morley PS, Dargatz DA, Hyatt DR, Dewell GA, Patterson JG, et al (2011) Effects of restricted antimicrobial exposure on antimicrobial resistance in fecal *Escherichia coli* from feedlot cattle. Foodborne Pathog Dis 8: 87–98. 10.1089/fpd.2010.0632 21034271

[17] Berge AC, Hancock DD, Sischo WM, Besser TE (2010) Geographic, farm, and animal factors associated with multiple antimicrobial resistance in fecal *Escherichia coli* isolates from cattle in the western United States. J Am Vet Med Assoc 236: 1338–1344. 10.2460/javma.236.12.1338 20550450

[18] Brooks JP, Adeli A, McLaughlin MR (2014) Microbial ecology, bacterial pathogens, and antibiotic resistant genes in swine manure wastewater as influenced by three swine management systems. Water Res 57C: 96–103. 10.1016/j.watres.2014.03.017 24704907

[19] Nagulapally SR, Ahmad A, Henry A, Marchin GL, Zurek L, et al (2009) Occurrence of ciprofloxacin-, trimethoprim-sulfamethoxazole-, and vancomycin-resistant bacteria in a municipal wastewater treatment plant. Water Environ Res 81: 82–90. 19280903 10.2175/106143008x304596

[20] Berge AC, Dueger EL, Sischo WM (2006) Comparison of *Salmonella enterica* serovar distribution and antibiotic resistance patterns in wastewater at municipal water treatment plants in two California cities. J Appl Microbiol 101: 1309–1316. 17105561 10.1111/j.1365-2672.2006.03031.x

[21] Pruden A, Arabi M, Storteboom HN (2012) Correlation between upstream human activities and riverine antibiotic resistance genes. Environ Sci Technol 46: 11541–11549. 10.1021/es302657r 23035771

[22] Durso LM, Miller DN, Wienhold BJ (2012) Distribution and quantification of antibiotic resistant genes and bacteria across agricultural and non-agricultural metagenomes. PLoS One 7: e48325 10.1371/journal.pone.0048325 23133629 PMC3487761

[23] Alm EW, Zimbler D, Callahan E, Plomaritis E (2014) Patterns and persistence of antibiotic resistance in faecal indicator bacteria from freshwater recreational beaches. J Appl Microbiol. 10.1111/jam.12512 24698413

[24] FDA (2003) Evaluating the Safety of Antimicrobial New Animal Drugs with Regard to Their Microbiological Effects on Bacteria of Human Health Concern.

[25] AGISAR (2012) Critically important antimicrobials for human medicine, Advisory Group on integrated surveillance of antimicrobial resistance (AGISAR), 3rd revision, World Health Organization (WHO), Geneva, Switzerland Available: http://apps.who.int/iris/bitstream/10665/77376/1/9789241504485_eng.pdf. Accessed 06.30.14.

[26] Daniels JB, Call DR, Hancock D, Sischo WM, Baker K, et al (2009) Role of ceftiofur in selection and dissemination of blaCMY-2-mediated cephalosporin resistance in Salmonella enterica and commensal Escherichia coli isolates from cattle. Appl Environ Microbiol 75: 3648–3655. 10.1128/AEM.02435-08 19376926 PMC2687309

[27] Frye JG, Jackson CR (2013) Genetic mechanisms of antimicrobial resistance identified in *Salmonella enterica*, *Escherichia coli*, and Enteroccocus spp. isolated from U.S. food animals. Front Microbiol 4: 135 10.3389/fmicb.2013.00135 23734150 PMC3661942

[28] Strahilevitz J, Jacoby GA, Hooper DC, Robicsek A (2009) Plasmid-mediated quinolone resistance: a multifaceted threat. Clin Microbiol Rev 22: 664–689. 10.1128/CMR.00016-09 19822894 PMC2772364

[29] Winokur PL, Brueggemann A, DeSalvo DL, Hoffmann L, Apley MD, et al (2000) Animal and human multidrug-resistant, cephalosporin-resistant *Salmonella* isolates expressing a plasmid-mediated CMY-2 AmpC beta-lactamase. Antimicrob Agents Chemother 44: 2777–2783. 10991860 10.1128/aac.44.10.2777-2783.2000 PMC90151

[30] Brichta-Harhay DM, Arthur TM, Bosilevac JM, Guerini MN, Kalchayanand N, et al (2007) Enumeration of *Salmonella* and *Escherichia coli* O157:H7 in ground beef, cattle carcass, hide and faecal samples using direct plating methods. J Appl Microbiol 103: 1657–1668. 17953577 10.1111/j.1365-2672.2007.03405.x

[31] Barkocy-Gallagher GA, Edwards KK, Nou X, Bosilevac JM, Arthur TM, et al (2005) Methods for recovering *Escherichia coli* O157:H7 from cattle fecal, hide, and carcass samples: sensitivity and improvements. J Food Prot 68: 2264–2268. 16300061 10.4315/0362-028x-68.11.2264

[32] Nou X, Arthur TM, Bosilevac JM, Brichta-Harhay DM, Guerini MN, et al (2006) Improvement of immunomagnetic separation for *Escherichia coli* O157:H7 detection by the PickPen magnetic particle separation device. J Food Prot 69: 2870–2874. 17186652 10.4315/0362-028x-69.12.2870

[33] Rahn K, De Grandis SA, Clarke RC, McEwen SA, Galan JE, et al (1992) Amplification of an *inv*A gene sequence of *Salmonella* Typhimurium by polymerase chain reaction as a specific method of detection of *Salmonella* . Mol Cell Probes 6: 271–279. 1528198 10.1016/0890-8508(92)90002-f

[34] Nucera DM, Maddox CW, Hoien-Dalen P, Weigel RM (2006) Comparison of API 20E and *inv*A PCR for identification of *Salmonella enterica* isolates from swine production units. J Clin Microbiol 44: 3388–3390. 16954281 10.1128/JCM.00972-06 PMC1594722

[35] Horakova K, Mlejnkova H, Mlejnek P (2008) Specific detection of *Escherichia coli* isolated from water samples using polymerase chain reaction targeting four genes: cytochrome bd complex, lactose permease, beta-D-glucuronidase, and beta-D-galactosidase. J Appl Microbiol 105: 970–976. 10.1111/j.1365-2672.2008.03838.x 18489560

[36] Deasy BM, Rea MC, Fitzgerald GF, Cogan TM, Beresford TP (2000) A rapid PCR based method to distinguish between *Lactococcus* and *Enterococcus* . Syst Appl Microbiol 23: 510–522. 11249021 10.1016/S0723-2020(00)80025-9

[37] Livak KJ, Schmittgen TD (2001) Analysis of relative gene expression data using real-time quantitative PCR and the 2(-Delta Delta C(T)) Method. Methods 25: 402–408. 11846609 10.1006/meth.2001.1262

[38] Hammer Ø, Harper DAT, Ryan PD (2001) PAST: Paleontological Statistics Software Package for Education and Data Analysis. Palaeontologia Electronica 4: 9.

[39] USDA-FDA (2009) Summary Report on Antimicrobials Sold or Distributed for Use in Food-Producing Animals.

[40] CDC (2013) Antibiotic Resistance Threats in the United States, 2013. Centers for Disease Control.

[41] Scott HM, Campbell LD, Harvey RB, Bischoff KM, Alali WQ, et al (2005) Patterns of antimicrobial resistance among commensal *Escherichia coli* isolated from integrated multi-site housing and worker cohorts of humans and swine. Foodborne Pathog Dis 2: 24–37. 15992296 10.1089/fpd.2005.2.24

[42] Aarestrup FM (2000) Occurrence, selection and spread of resistance to antimicrobial agents used for growth promotion for food animals in Denmark. APMIS Suppl 101: 1–48. 11125553

[43] Agerso Y, Aarestrup FM, Pedersen K, Seyfarth AM, Struve T, et al (2012) Prevalence of extended-spectrum cephalosporinase (ESC)-producing *Escherichia coli* in Danish slaughter pigs and retail meat identified by selective enrichment and association with cephalosporin usage. J Antimicrob Chemother 67: 582–588. 10.1093/jac/dkr507 22207594

[44] Singer RS, Ward MP, Maldonado G (2006) Can landscape ecology untangle the complexity of antibiotic resistance? Nat Rev Microbiol 4: 943–952. 17109031 10.1038/nrmicro1553

[45] Alroy K, Ellis JC (2011) Pilot study of antimicrobial-resistant *Escherichia coli* in herring gulls (Larus argentatus) and wastewater in the northeastern United States. J Zoo Wildl Med 42: 160–163. 22946391 10.1638/2010-0130.1

[46] Normand EH, Gibson NR, Reid SW, Carmichael S, Taylor DJ (2000) Antimicrobial-resistance trends in bacterial isolates from companion-animal community practice in the UK. Prev Vet Med 46: 267–278. 10960713 10.1016/s0167-5877(00)00149-5

[47] Sayah RS, Kaneene JB, Johnson Y, Miller R (2005) Patterns of antimicrobial resistance observed in *Escherichia coli* isolates obtained from domestic- and wild-animal fecal samples, human septage, and surface water. Appl Environ Microbiol 71: 1394–1404. 15746342 10.1128/AEM.71.3.1394-1404.2005 PMC1065171

[48] Ibekwe AM, Murinda SE, Graves AK (2011) Genetic diversity and antimicrobial resistance of *Escherichia coli* from human and animal sources uncovers multiple resistances from human sources. PLoS One 6: e20819 10.1371/journal.pone.0020819 21687635 PMC3110821

[49] Pruden A, Larsson DG, Amezquita A, Collignon P, Brandt KK, et al (2013) Management options for reducing the release of antibiotics and antibiotic resistance genes to the environment. Environ Health Perspect 121: 878–885. 10.1289/ehp.1206446 23735422 PMC3734499

[50] Miteva VI, Sheridan PP, Brenchley JE (2004) Phylogenetic and physiological diversity of microorganisms isolated from a deep greenland glacier ice core. Appl Environ Microbiol 70: 202–213. 14711643 10.1128/AEM.70.1.202-213.2004 PMC321287

[51] Brown MG, Balkwill DL (2009) Antibiotic resistance in bacteria isolated from the deep terrestrial subsurface. Microb Ecol 57: 484–493. 10.1007/s00248-008-9431-6 18677528

[52] Bhullar K, Waglechner N, Pawlowski A, Koteva K, Banks ED, et al (2012) Antibiotic resistance is prevalent in an isolated cave microbiome. PLoS One 7: e34953 10.1371/journal.pone.0034953 22509370 PMC3324550

[53] Davis CE, Anandan J (1970) The evolution of r factor. A study of a "preantibiotic" community in Borneo. N Engl J Med 282: 117–122. 4902658 10.1056/NEJM197001152820302

[54] Bartoloni A, Pallecchi L, Rodriguez H, Fernandez C, Mantella A, et al (2009) Antibiotic resistance in a very remote Amazonas community. Int J Antimicrob Agents 33: 125–129. 10.1016/j.ijantimicag.2008.07.029 18947984

[55] Bartoloni A, Bartalesi F, Mantella A, Dell'Amico E, Roselli M, et al (2004) High prevalence of acquired antimicrobial resistance unrelated to heavy antimicrobial consumption. J Infect Dis 189: 1291–1294. 15031799 10.1086/382191

[56] Iwane T, Urase T, Yamamoto K (2001) Possible impact of treated wastewater discharge on incidence of antibiotic resistant bacteria in river water. Water Sci Technol 43: 91–99. 11380211

[57] Korzeniewska E, Korzeniewska A, Harnisz M (2013) Antibiotic resistant *Escherichia coli* in hospital and municipal sewage and their emission to the environment. Ecotoxicol Environ Saf 91: 96–102. 10.1016/j.ecoenv.2013.01.014 23433837

[58] Luczkiewicz A, Jankowska K, Bray R, Kulbat E, Quant B, et al (2011) Antimicrobial resistance of fecal indicators in disinfected wastewater. Water Sci Technol 64: 2352–2361. 10.2166/wst.2011.769 22170827

[59] Novo A, Andre S, Viana P, Nunes OC, Manaia CM (2013) Antibiotic resistance, antimicrobial residues and bacterial community composition in urban wastewater. Water Res 47: 1875–1887. 10.1016/j.watres.2013.01.010 23375783

[60] Reinthaler FF, Posch J, Feierl G, Wust G, Haas D, et al (2003) Antibiotic resistance of *E*. *coli* in sewage and sludge. Water Res 37: 1685–1690. 12697213 10.1016/S0043-1354(02)00569-9

[61] Rizzo L, Manaia C, Merlin C, Schwartz T, Dagot C, et al (2013) Urban wastewater treatment plants as hotspots for antibiotic resistant bacteria and genes spread into the environment: a review. Sci Total Environ 447: 345–360. 10.1016/j.scitotenv.2013.01.032 23396083

[62] Kummerer K (2004) Resistance in the environment. J Antimicrob Chemother 54: 311–320. 15215223 10.1093/jac/dkh325

[63] Kummerer K, Henninger A (2003) Promoting resistance by the emission of antibiotics from hospitals and households into effluent. Clin Microbiol Infect 9: 1203–1214. 14686985 10.1111/j.1469-0691.2003.00739.x

[64] Novais C, Coque TM, Ferreira H, Sousa JC, Peixe L (2005) Environmental contamination with vancomycin-resistant enterococci from hospital sewage in Portugal. Appl Environ Microbiol 71: 3364–3368. 15933043 10.1128/AEM.71.6.3364-3368.2005 PMC1151839

[65] Zhang Y, Marrs CF, Simon C, Xi C (2009) Wastewater treatment contributes to selective increase of antibiotic resistance among Acinetobacter spp. Sci Total Environ 407: 3702–3706. 10.1016/j.scitotenv.2009.02.013 19321192

[66] Brown KD, Kulis J, Thomson B, Chapman TH, Mawhinney DB (2006) Occurrence of antibiotics in hospital, residential, and dairy effluent, municipal wastewater, and the Rio Grande in New Mexico. Sci Total Environ 366: 772–783. 16313947 10.1016/j.scitotenv.2005.10.007

[67] Harris S, Morris C, Morris D, Cormican M, Cummins E (2014) Antimicrobial resistant *Escherichia coli* in the municipal wastewater system: effect of hospital effluent and environmental fate. Sci Total Environ 468–469: 1078–1085. 10.1016/j.scitotenv.2013.09.017 24100208

[68] Nesme J, Cecillon S, Delmont TO, Monier JM, Vogel TM, et al (2014) Large-scale metagenomic-based study of antibiotic resistance in the environment. Curr Biol 24: 1096–1100. 10.1016/j.cub.2014.03.036 24814145

[69] Mather AE, Reid SW, Maskell DJ, Parkhill J, Fookes MC, et al (2013) Distinguishable epidemics of multidrug-resistant *Salmonella* Typhimurium DT104 in different hosts. Science 341: 1514–1517. 10.1126/science.1240578 24030491 PMC4012302

[70] Threlfall EJ, Day M, de Pinna E, Charlett A, Goodyear KL (2006) Assessment of factors contributing to changes in the incidence of antimicrobial drug resistance in *Salmonella enterica* serotypes Enteritidis and Typhimurium from humans in England and Wales in 2000, 2002 and 2004. Int J Antimicrob Agents 28: 389–395. 17029756 10.1016/j.ijantimicag.2006.07.009

[71] Cox R, Su T, Clough H, Woodward MJ, Sherlock C (2012) Spatial and temporal patterns in antimicrobial resistance of *Salmonella* Typhimurium in cattle in England and Wales. Epidemiol Infect 140: 2062–2073. 22214772 10.1017/S0950268811002755

[72] Lowrance TC, Loneragan GH, Kunze DJ, Platt TM, Ives SE, et al (2007) Changes in antimicrobial susceptibility in a population of *Escherichia coli* isolated from feedlot cattle administered ceftiofur crystalline-free acid. Am J Vet Res 68: 501–507. 17472449 10.2460/ajvr.68.5.501

[73] Singer RS, Patterson SK, Wallace RL (2008) Effects of therapeutic ceftiofur administration to dairy cattle on *Escherichia coli* dynamics in the intestinal tract. Appl Environ Microbiol 74: 6956–6962. 10.1128/AEM.01241-08 18820057 PMC2583494

