# Supplementary figures and images for: Antimicrobial-Resistant Bacterial Populations and Antimicrobial Resistance Genes Obtained from Environments Impacted by Livestock and Municipal Waste
Source: PLoS One. 2015 Jul 21;10(7):e0132586. doi: 10.1371/journal.pone.0132586 (PMC4510610; doi:10.1371/journal.pone.0132586)

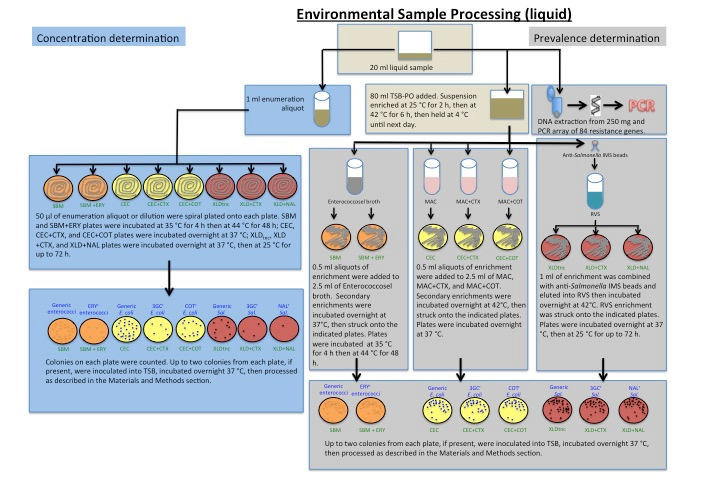

Supplement: S1 Fig — (TIF) [file pone.0132586.s001.tif]

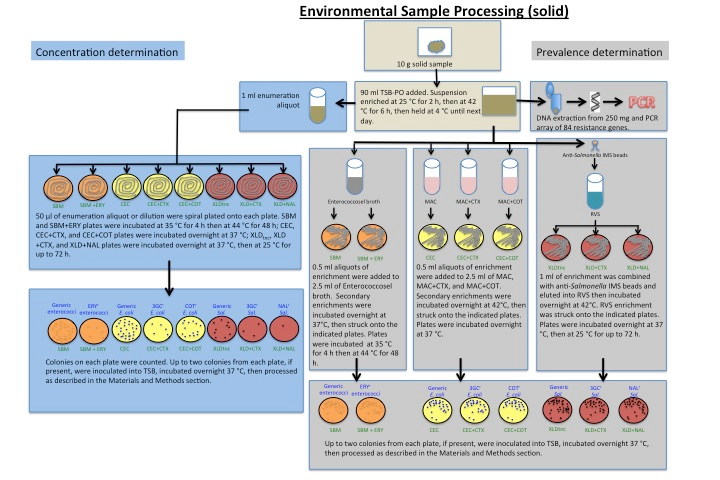

Supplement: S2 Fig — (TIF) [file pone.0132586.s002.tif]
