# Supplementary material for: Antimicrobial-Resistant Bacterial Populations and Antimicrobial Resistance Genes Obtained from Environments Impacted by Livestock and Municipal Waste
Source: PLoS One. 2015 Jul 21;10(7):e0132586. doi: 10.1371/journal.pone.0132586 (PMC4510610; doi:10.1371/journal.pone.0132586)
Supplement: S1 Table — The file contains data from relative quantitation of AMR genes. Comparisons were between municipal and cattle samples and municipal and swine samples. (DOCX) [file pone.0132586.s003.docx]

**Table S1. Relative abundance of antimicrobial resistance genes in livestock and municipal environments.**

|  | Log10-fold change | |
| --- | --- | --- |
| Gene designation | Municipal/Cattle | Municipal/Swine |
| OXA-10 Group | 2.96 | 2.44 |
| AAC(6)-Ib-cr | 2.53 | 1.05 |
| OXA-2 Group | 1.69 | 1.02 |
| *erm*B | 1.46 | 0.50 |
| *tet*A | 0.88 | 0.46 |
| *aad*A1 | 0.20 | 0.23 |
| *mef*A | 0.06 | 0.48 |
